# Bacterial actin MreB as a new antibiotic target

**DOI:** 10.3389/fmolb.2026.1912759

**Published:** 2026-10-01

**Authors:** Emőke Bódis, Beáta Longauer, András Lukács, Tamás Huber, Miklós Nyitrai, Géza Makkai, Tibor Jánosi, Rita Börzsei, Csaba Hetényi, Zoltán Gazdag, Szilvia Barkó

**Affiliations:** 1 Department of Biophysics, Medical School, University of Pécs, Pécs, Hungary; 2 Nano-Bio-Imaging Core Facility, Medical School, University of Pécs, Pécs, Hungary; 3 Department of Pharmacology and Pharmacotherapy, Pharmacoinformatics Unit Medical School, University of Pécs, Pécs, Hungary; 4 Department of Molecular Biology and Microbiology Faculty of Sciences, University of Pécs, Pécs, Hungary

**Keywords:** A22, minimal inhibitory concentration, MreB cytoskeleton, spectroscopy, structured illumination microscope, synergism, vancomycin

## Abstract

Actin homolog MreB plays a crucial role in the synthesis of the bacterial cell wall. Disruption of the MreB filament network results in the disruption of the cell wall, which thus serves an unconventional antibacterial strategy. A22, a small inhibitor molecule, binds directly to the MreB filament, altering its structure and causing a morphological change of the cell, leading to cell death. This effective antibacterial property is shadowed by the fact that at high concentration, A22 is toxic to eukaryotic host cells, so it cannot be administered at higher doses. Vancomycin is a widely used antibiotic targeting Gram-positive bacteria at concentrations as high as 10–20 μg/mL, which can occasionally cause serious side effects. In this study, we present our recent findings that a synergistic interaction exists between A22 and vancomycin, which results in the inhibition of cell proliferation by the combined use of very low concentrations of A22 (6.25 μg/mL) and vancomycin (1.56 μg/mL). In addition, in the presence of A22, vancomycin is effective not only against Gram-positive bacteria but also against Gram-negative bacteria. Since 5 of the 7 most common antibiotic-resistant bacteria according to the WHO are Gram-negative, this synergism between A22 and vancomycin opens a new way of combating bacterial infections. Our spectroscopic results indicate that vancomycin binds directly to MreB; using a computer docking method, we determined the possible binding site of vancomycin on the MreB filament. This newly identified interaction between vancomycin and A22 highlights the potential of the combined use, providing a new strategy to tackle antibiotic resistance.

## 1 Introduction

The bacterial actin homolog MreB plays a fundamental role in forming the cell wall and maintaining basic cellular functions, like cell division, chromosome segregation, cell wall polarity, and cell wall morphogenesis (White and Gober, 2012; Strahl et al., 2014). Even though the structure of monomeric MreB shows strong similarity to that of eukaryotic actin, the two filaments differ greatly from each other. While actin protofilaments are parallel and twisted, MreB filaments are antiparallel and straight (Ent et al., 2014). Gram-positive bacteria have multiple MreB homologs, while Gram-negative bacteria have a single *mreB* gene; therefore, the knockout of MreB function could be lethal for Gram-negative bacteria (Levin et al., 1992; Varley and Stewart, 1992). This explains the increase in the number of publications focusing on MreB as a potential antibiotic target (Awuni, 2020; Bryan et al., 2022; Sagong et al., 2022). The fact that actin and MreB have different filamental structures could be a significant advantage in the fight against pathogenic bacteria. This makes it possible to develop specific bacterial inhibitors that do not have any toxic effect on mammalian cells.

A22 (S-(3,4-dichlorobenzyl) isothiourea) has been found as a toxin specifically against MreB, which can be that specifically targets exclusively to Gram-negative bacteria (Bean et al., 2009). The binding site of A22 is adjacent to the nucleotide-binding site; thus, A22 binding can block the release of P_i_ from the ATP-binding pocket, resulting in the destabilization of the filament (Awuni et al., 2016). Without proper ATP binding and hydrolysis, MreB cannot maintain the necessary structure required for polymerization. Although the toxicity of A22 in eukaryotic cells has been reported, severe contradictions can be found in the literature regarding the mechanism and required concentrations. It was proved that A22 can bind directly to actin, which affects cell growth and shows cytotoxic effects. It is also reported that different cell types react with varying sensitivity to the presence of A22. For example, A22 shows minimal cytotoxic activity against peripheral blood mononuclear cells (Bonez et al., 2016; Kotzialampou et al., 2021), but it is toxic to glioblastoma cell lines (Kumar et al., 2024). Since the mechanism of action of A22 could open up novel paths against pathogenic bacteria, intensive research is being conducted in this area (Muheim et al., 2017; Kumar et al., 2024), even in cases of multidrug-resistant bacteria (Nicholson et al., 2012).

A combination of different antibacterial agents, e.g., the use of clinical antibiotics with anti-MreB compounds or adjuvants, can tackle the growing problem of multidrug resistance. One very beneficial approach is to use different antibiotics in combination with A22 to achieve greater antibacterial efficacy (Kotzialampou et al., 2021) through destroying the MreB filamental structure, which has a multifunctional character and therefore is essential for bacterial viability. For example, synergism occurred in the case of A22-cefoxitin and A22-azithromycin against *Escherichia coli* (Kotzialampou et al., 2021). This synergistic effect has not yet been tested or described for dual A22-vancomycin use.

Vancomycin is an antibiotic used to treat Gram-positive bacterial infections. This tricyclic glycopeptide inhibits cell wall formation in streptococci, enterococci, and staphylococci (Kang and Park, 2015). To achieve optimal results when administering vancomycin to patients, dosage adjustment is based on patient-specific factors. The typical dosage is 125 mg–1 g in vial as recommended by the WHO (World Health Organization, 2025). Intravenous vancomycin dosing typically achieves steady-state trough serum concentrations of approximately 10–20 μg/mL in adults with normal renal function (James and Gurk-Turner, 2001; Kitzis and Goldstein, 2006; Perin et al., 2020). In the case of infections caused by Gram-negative bacteria, vancomycin treatment is not considered as the minimal inhibitory concentration (MIC) of vancomycin is much higher for Gram-negative bacteria (200–250 μg/mL). Even vancomycin-resistant strains have a much lower MIC value (8 μg/mL, Vancomycin-resistant *Staphylococcus aureus*). The side effects of vancomycin (hypersensitivity reactions, nephrotoxicity, and ototoxicity) occur even at the indicated concentration, so it is not possible to increase the dose further.

Our experiments show, however, that when the MreB inhibitor A22 is used in combination with vancomycin, the resulting powerful synergy causes the MIC value for Gram-negative bacteria to reach that of Gram-positive bacteria. In other words, A22 makes Gram-negative bacteria “Gram-positive-like” in terms of their resistance to vancomycin.

The ESKAPEE group is an acronym for antibiotic-resistant bacteria responsible for hospital-acquired infections (Krul et al., 2025). They pose the greatest threat to global health and healthcare systems because they are difficult to treat due to their resistance to drugs. The members of the group (*Enterococcus faecium*, *Staphylococcus aureus*, *Klebsiella pneumoniae*, *Acinetobacter baumannii*, *Pseudomonas aeruginosa*, *Enterobacter* species, and *E. coli*) (except for the first two) are Gram-negative, for which vancomycin treatment based on the synergism with A22 may be an effective tool.

As we mentioned, our microbiological and microscopic measurements showed the synergistic effect between A22 and vancomycin. Furthermore, we confirmed that vancomycin binds directly to the MreB protein *in vivo* and presumably induces structural changes in it. This statement was further strengthened by finding the putative binding sites of vancomycin on MreB by molecular docking calculations. Our findings may provide a basis for research aimed at developing drugs—through the combined action of one or even several antibiotics—that inhibit bacterial growth by blocking the function of the MreB protein, especially in Gram-negative infections.

## 2 Materials and methods

### 2.1 Determination of minimal inhibitory concentration and synergism

Minimal inhibitory concentration (MIC) values were determined according to the Clinical and Laboratory Standards Institute (CLSI) M07-A9 broth microdilution method (Clinical and Laboratory Standards Institute, 2015). A22 was dissolved in Dimethyl sulfoxide, and vancomycin was dissolved in distilled water. The antibacterial agents were tested in a 96-well plate using twofold serial dilutions ranging from 0.78 to 400 μg mL^−1^ for vancomycin and 1.56–200 μg mL^−1^ for A22. The final solvent concentration for each treatment was 1% (v/v). The optical density of the bacterial suspension was set to 0.12 (OD_625_, McFarland standard 0.5), and a 500-fold dilution with LB Broth (Liquid microbial growth medium) nutrient solution was used to obtain a concentration of 2∙10^5^ cells/mL. The results were evaluated after 24 h of incubation at 37 °C. Following treatment, cell densities were measured by a Multiskan EX 355 (Thermo Electron Corporation) spectrophotometer at a wavelength of 594 nm. The evaluation of 80% inhibitory effects (MIC_80_) of the antibacterial agents was applied to and compared with antibacterial agent-free controls. Each treatment was performed in triplicate.

The interaction between vancomycin and A22 against the *E. coli* K12 strain was examined with the chequerboard broth microdilution method. Sample preparation for microdilution was performed according to the antibacterial susceptibility method as described above. The antibacterial agent’s interaction types were determined based on the fractional inhibitory concentration index (FICI) calculated according to Nyilasi et al. (2014). Using the lowest MIC value of the antimicrobial agents, the value of the fractional inhibitory concentration index, i.e., ƩFICI (fractional inhibitory concentration index), was calculated using the formula below. If ƩFICI ≤ 0.5, this implies synergism, and the two antibacterial agents support the effect of each other: The FICI value can be calculated with Equation 1.

$$ƩFICI=\frac{{MIC}_{A,together}}{{MIC}_{A,itself}}+\frac{{MIC}_{B,together}}{{MIC}_{B,itself}}.$$ (1)

### 2.2 Examination of the growth curve

*E. coli* K12 cell cultures (Sigma-Aldrich) were grown overnight in Luria Broth (0.5% yeast extract, 1% NaCl, and 1% peptone) nutrient solution at 37 °C in a shaking incubator. The next day, in the absence or presence of 1.56 μg/mL vancomycin and/or 6.25 μg/mL A22, a 100-fold dilution of the bacterial culture was made, which was grown in 5 mL of fresh nutrient solution. The optical density (OD) of the cells was detected repeatedly at 600 nm using a Perkin Elmer Lambda XLS+ spectrophotometer. We diluted the cultures to a value above an optical density of 0.5, and then measured the OD value.

### 2.3 Visualization of BODIPY-vancomycin uptake in *E. coli* cells

*E. coli* K12 cells were grown overnight at 37 °C in LB medium in a shaking incubator. The following day, we started the culture from this sample, making a 100-fold dilution. Additionally, a 2% agarose pad was prepared by dissolving 2 g of agarose in 100 mL of LB medium. Agarose was dissolved by heating in a microwave for 30–60 s. Then, 100 μg/mL vancomycin or 27 μg/mL A22, both, or neither was added to the fluid agarose. Finally, 1 μg/mL BODIPY-vancomycin was applied to each sample. When the agarose solidified on the coverslip and the cultures reached the logarithmic phase after 200 min, the cells were smeared on an agarose pad. After an additional 2 h incubation period, the cells were visualized using an Olympus IX81 microscope. The percentage of cells that took BODIPY-vancomycin was determined by combining the transmission and fluorescence images. The percentages of cultures containing either vancomycin or vancomycin and A22 were subjected to a t-test to determine whether the difference in response to A22 treatment was significant.

### 2.4 Fluorescence microscopy observations

*E. coli* K12 cells were grown for 3 h as described above (see Section 2.2). The cells were harvested by centrifugation at 15,000 g at 22 °C for 1 min. After washing in Phosphate-Buffered Saline (PBS), the pellet was resuspended in paraformaldehyde solution (4%) and stored at room temperature for 10 min. Following another wash, the samples were labeled with BODIPY-vancomycin (1 μM = 1.49 μg/mL). The cells were centrifuged, and after a washing step in PBS, they were applied to a poly-L-lysine-coated coverslip. The cells were visualized with Zeiss Elyra S1 structured illumination microscopy (SIM), and images were analyzed using Fiji software.

### 2.5 Investigation of binding between *Tm*-MreB and BODIPY-vancomycin

The expression and purification of *Thermotoga maritima*-MreB (*Tm*-MreB) protein were achieved as described earlier (Longauer et al., 2022).

### 2.6 Steady-state anisotropy measurements

Steady-state anisotropy measurements were performed using a Jobin Yvon Horiba fluorimeter. Binding was tested using 1 μM fluorescently labeled vancomycin (Invitrogen) (BODIPY™ FL Vancomycin, FLV), with the protein concentration gradually increased from 1 to 60 µM. Our test was performed with a 0.2 mM ATP and/or polymerizing salt (100 mM KCl and 2 mM MgCl_2_). The following equation was used to determine the binding affinity of BODIPY-vancomycin to *Tm-*MreB: The binding affinity was calculated by Equation 2.

$$y=\frac{y_{\min}+\left(\left(y_{\max}-y_{\min}\right)\right.*\left(\left(\left(KD+FLV+x\right)-{\left((\left(KD+FLV+x\right)\right.}^{2})-{\left.\left(4*FLV*x\right)\right)}^{0,5}\right)\right)}{\left(2*FLV\right)}.$$ (2)

In this equation, the following values were used: *x* (*Tm*-MreB concentration), *y*_*min*_ (the lowest fluorescence anisotropy value), *y*_*max*_ (the highest saturation fluorescence anisotropy value), *FLV* (the concentration of fluorescent vancomycin), and *KD* (the binding constant).

### 2.7 Sedimentation assay

In the presence or absence of 100 µg/mL of unlabeled vancomycin, 20 μM *Tm*-MreB was incubated overnight at 4 °C. The next day, the samples were ultracentrifuged at 100,000 × *g* at 22 °C for 30 min. The pellets and supernatants were analyzed by 10% SDS-PAGE.

### 2.8 Labeling of *Tm*-MreB with BODIPY-vancomycin

In the presence or absence of 100 μg/mL of native vancomycin, 20 μM of *Thermotoga maritima*-MreB (*Tm-*MreB) was incubated for 2 h with 1 μΜ (=1.45 μg/mL) of BODIPY-vancomycin. Samples were then dropped onto coverslips and examined at ×100 magnification using an Olympus IX 81 fluorescence microscope.

### 2.9 Structural calculations

#### 2.9.1 Target preparation

The apo structure of the *Tm-*MreB protein (PDB code: 1jce (Ent et al., 2001)) was obtained from the PDB database (Berman et al., 2000). Water molecules were deleted, hydrogen atoms were added to the structure, and the N-terminal of the protein was neutralized by capping with the methyl-acetate group (ACE). This structure was used as the ligand-free (apo) monomeric *Tm-*MreB. Symmetry mates of the apo *Tm-*MreB were generated by PyMol (Schrödinger, 2015) to use the linear dimer structure (Ent et al., 2001) for calculations.

#### 2.9.2 Ligand preparation

The crystal structure of the vancomycin dimer was obtained from the PDB database (PDB code: 1sho (Schäfer et al., 1996)). Water and ion molecules were removed, and hydrogen atoms were added to the structure. The molecule was energy-minimized using the semiempirical quantum chemistry program package, MOPAC (Stewart, 2013), with PM7 parameterization (Stewart, 2013). The minimized monomeric form was then used for docking calculations.

#### 2.9.3 Vancomycin dimer

The minimized crystallographic dimeric vancomycin (PDB code: 1sho (Schäfer et al., 1996)) was superimposed on the representative monomer conformation (rank 1) docked to the dimer target structure using the pair fitting module of PyMol (Schrödinger, 2015), resulting in 0.377 Cα root-mean-square deviation (RMSD). The pdbqt files for the representative docked monomers and the opposite partner of the aligned dimeric structures were prepared by AutoDock Tools (Morris et al., 2009) using torsion restraints. The pdbqt files were merged to produce the dimeric vancomycin in pdbqt format. This dimeric ligand structure was used in docking calculations.

#### 2.9.4 Docking calculations

The minimized and aligned vancomycin ligands were subjected to docking calculations using AutoDock 4 (Morris et al., 2009). Gasteiger–Marsili partial charges were assigned to both the ligand and MreB target structures in AutoDock Tools (Morris et al., 2009), and a united-atom representation was applied for non-polar moieties. In all calculations, targets were treated rigidly. Flexibility was allowed for all active torsions of monomeric vancomycin; however, torsion restraints were applied to the dimeric form. Grid maps were prepared by AutoGrid4 (Morris et al., 2009), centered at 27.99, 20.69, and 21.15 and 31.99, 24.69, and 21.15 with 100 × 100 × 100 and 126 × 126 × 126 grid points at a spacing of 0.375 Å for monomeric and dimeric *Tm-*MreBs, respectively. A Lamarckian genetic algorithm was used for global search in 100 docking runs covering both the dimerization interface (Ent et al., 2001) and the ATP-binding site (Ent et al., 2001). Docked ligand conformations were ranked based on corresponding calculated target–ligand intermolecular interaction energy (E_inter_) values and subsequently clustered using a root-mean-square deviation (RMSD) tolerance of 3.5 Å between cluster members. E_inter_ is the sum of van der Waals, H-bond, desolvation, and electrostatic interaction energies (Huey et al., 2007). Conformations were selected and studied further based on the docking locations and interaction energy values (i.e., rank 1, rank 2, rank 22, and rank 52).

#### 2.9.5 Calculation of the stabilizing energy contribution of vancomycin for *Tm*-MreB dimerization

Vancomycin’s role for *Tm-*MreB dimerization was also examined from an energetical point of view. E_inter_ values between the MreB monomers were calculated by using the “epdb” keyword in the dpf file of AutoDock (Huey et al., 2007). First, E_inter_ was calculated between two vancomycin-free *Tm-*MreB monomer units and named as E_dimer_(apo). Second, E_inter_ was calculated between a vancomycin-bound *Tm-*MreB monomer unit and a vancomycin-free *Tm-*MreB monomer unit, and it was named as E_dimer_(holo). The stabilizing effect of vancomycin on the dimerization of *Tm-*MreB monomer units was expressed as the stabilizing energy contribution of vancomycin (E_st_) using the percentage of E_inter_(apo) calculated by Equation 3. E_st_ shows the energy gain of *Tm-*MreB dimerization in the presence of vancomycin compared with the absence of it expressed in percentage:

$$E_{st}=\frac{E_{dimer}\left(holo\right)-E_{dimer}\left(apo\right)}{E_{dimer}\left(apo\right)}\times 100\;\%.$$ (3)

## 3 Results

The aim of this study was to investigate the presumed synergistic effect of the antibiotics A22 and vancomycin on MreB filaments inside *E. coli* cells and to determine how much they can enhance each other’s inhibitory effect as inferred from growth rate measurements. We also provide applicable concentrations of the antibiotics considering their synergistic effects. To determine the most probable binding sites of these antibiotics, *in silico* docking calculations were performed. Besides, using fluorescence spectroscopy and microscopy techniques, we provide strong evidence for the direct binding of vancomycin to MreB filaments.

### 3.1 Measurements on *E. coli* cells

#### 3.1.1 Microbiological FICI assay

Microbiological assays were used to determine the MIC_80_ (minimal inhibitory concentration) value of the two antimicrobials, which is the lowest concentration at which growth inhibition of more than 80% is observed. The synergistic effect between the two agents was detected by a checkerboard microdilution test, where FICI (fractional inhibitory concentration index) values under 0.5 represent synergism.

Based on our earlier microbiological data, our aim was to determine the lowest concentration of A22 and vancomycin that synergistically inhibits the bacterial growth of *E. coli* (Table 1). For this one, in a 96-well plate, various concentrations of A22 (1.56–200 μg/mL) and vancomycin (0.78–400 μg/mL) were added to 2^.^10^5^ cells/mL solution. As controls, cell growth inhibition was determined by A22 and vancomycin separately and in the absence of both. The inhibitory concentrations of A22 and vancomycin were 100 and 200 μg/mL, respectively. When the combination of A22 and vancomycin was applied, cell growth inhibition was observed in the range of 6.25–25 and 1.56–50 μg/mL, respectively (Table 1). Another method to demonstrate synergism between the two agents is to keep one agent constant while varying the concentration of the other. Accordingly, the inhibitory effect of vancomycin falls in the range of 6.25–50 μg/mL at a constant 3.125 μg/mL A22 concentration (Table 1).

**TABLE 1:** There is a synergistic effect between vancomycin and A22.

MIC_80_ alone (μg/mL)		MIC_80_ in combination (μg/mL)	FICI
A22 [100]	Van [200]	50 + 0.78	0.5
		6.25 + 1.56	0.07
		3.125 + 6.25	0.06
		1.56 + 25	0.14

The numbers in the left column refer to the MIC values for the two compounds separately. The middle column shows the concentration ranges in which the two antibiotics must be used to achieve a synergistic effect. If one of the antibiotics is kept constant, the concentration range of the other one changes to achieve the synergistic effect. The right column shows the fractional inhibitory concentration index (FICI) values of A22 and vancomycin in different combinations. FICI values below 0.5 mean that there is synergism between the two compounds.

The synergistic effect occurs at rather low vancomycin (1.56 μg/mL) and A22 (6.25 μg/mL) concentrations compared with the doses usually used in clinics (James and Gurk-Turner, 2001; Martin et al., 2010; Perin et al., 2020), and the combined use of an antimicrobial agent based on MreB inhibition may be suitable for Gram-negative infections.

#### 3.1.2 Growth rate of bacterial culture assays

For the determination of the growth rate of bacterial cultures, *E. coli* K12 cells were grown in the presence of 1.56 μg/mL of vancomycin and/or 6.25 μg/mL of A22 (these are the lowest synergistic inhibitory concentrations determined from the microbiological assay). As controls, cells were grown in the absence of any antibiotics (Figure 1A). Compared with previous literature data, these concentrations of both antibiotics are far below the values that would be toxic to human cells (for A22, it is between 25 and 32 μg/mL (Bonez et al., 2016; Kumar et al., 2024); for vancomycin, it is 2–5 mg/mL (Braun et al., 2020; Hall et al., 2024)).

**FIGURE 1:**
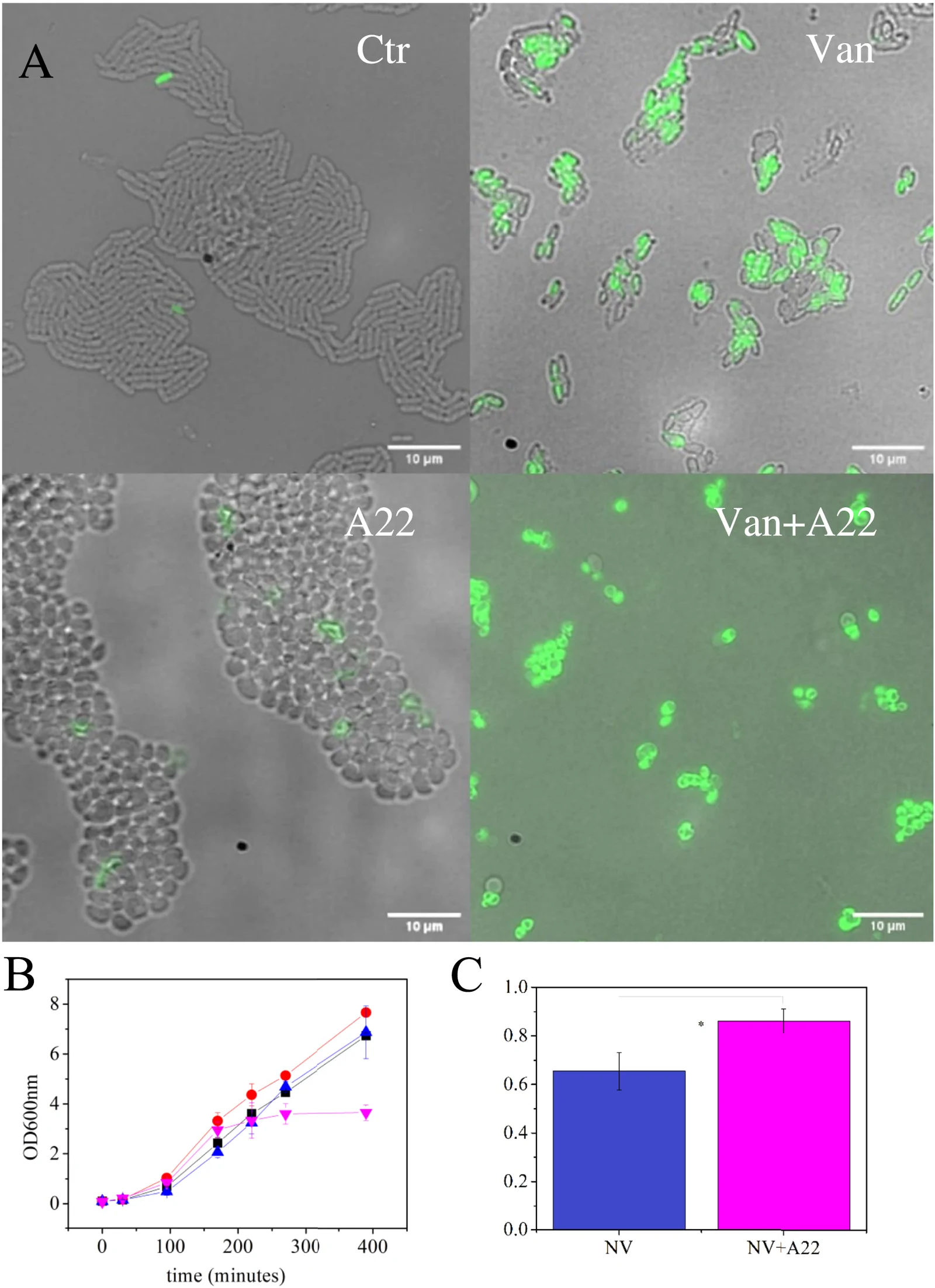
**(A)** Percentage of fluorescent cells increased with vancomycin and A22 treatment. *E. coli* K12 cells were grown in a BODIPY-vancomycin-containing agarose pad with or without 27 μg/mL A22, 100 μg/mL vancomycin, or both A22 and vancomycin at the specified amounts (ctr: control-without any antibiotics, Van: only vancomycin is present, A22: only A22 is present, and Van + A22: both vancomycin and A22 are applied). Samples were visualized using a fluorescence microscope with ×100 magnification. The transmission and fluorescence images were merged. Unlabeled vancomycin enhanced the entrance of BODIPY-vancomycin in *E. coli* cells, although A22 does not have this effect on its own. When A22 and vancomycin were present simultaneously, the uptake of BODIPY-vancomycin increased further. **(B)** *E. coli* growth was inhibited at the lowest antibiotic concentrations used in the FICI assay. *E. coli* K12 cells were grown in the absence of antibiotics (black squares), or in the presence of 6.25 μg/mL A22 (red circles), 1.56 μg/mL vancomycin (blue triangles), or both (magenta stars). For optical density values above 0.5, samples were diluted for measuring and calculating their original OD. The figure shows the standard deviation based on two sets of measurements. **(C)** In samples containing only vancomycin (blue column) or vancomycin and A22 (magenta column), the proportion of cells that took up fluorescent vancomycin was significantly different.

Our results showed that none of the antibiotics could separately inhibit the growth of bacteria at these applied concentration values (Figure 1B), and the tendency of bacterial growth is not different from that of the untreated cells. However, using vancomycin and A22 simultaneously, a strong inhibition of bacterial growth was observed (Figure 1B, magenta symbols). Based on this observation, vancomycin and A22 demonstrate strong synergy against *E. coli* growth.

#### 3.1.3 Fluorescence microscopy assay of vancomycin uptake

In order to determine whether the synergetic effect between A22 and vancomycin found in the microbiological assay could be explained by an increased rate of vancomycin entry into the cells, fluorescent microscopy assays were performed. *E. coli* K12 cells were grown in the presence of BODIPY-vancomycin in the presence and absence of vancomycin and A22. The existence of BODIPY-vancomycin in the growing medium (agarose pad) is needed for visualization using a transmission and fluorescence channel. Our results showed that in control experiments (in the absence of A22 and vancomycin), BODIPY-vancomycin hardly enters the cells (Figure 1C, upper left). The presence of A22 dissolved in the agarose pad did not enhance the appearance of BODIPY-vancomycin in the cells (Figure 1C, lower left). In contrast, the presence of native vancomycin significantly facilitated the cellular uptake of BODIPY-vancomycin (Figure 1C, upper right). The cells growing on the agarose pads containing both A22 and vancomycin took up an even higher proportion of BODIPY-vancomycin compared with the sample having only vancomycin or A22 (Figure 1B). To verify this observation, we performed a two-sample t-test using the percentage of labeled cells relative to the total number of the cells. A significant difference between the samples containing vancomycin and those containing vancomycin and A22 (Figure 1C) was observed. This finding suggests that A22 enhances vancomycin penetration into bacterial cells.

#### 3.1.4 Determination of the BODIPY-vancomycin localization in cells

Structured illumination microscopy (SIM) was used to determine whether the BODIPY-vancomycin labeling pattern in *E. coli* was localized intracellularly. If only the membrane or cell wall is labeled, no fluorescent labeling is expected in the cytoplasm (Supplementary Figure S1). However, if MreB binds BODIPY-vancomycin, intracellular labeling is expected. For this purpose, we examined cell membrane labeling with Nile Red fluorophore, which is known to give a signal in the apolar medium of the membrane. In this case, only the membrane gave a signal, and the interior of the cells remained unlabeled (Supplementary Figure S1). Comparison of the Nile Red- and the BODIPY-vancomycin-labeled cells highlights differences in the spatial distribution of fluorescence (Figure 2A). In the experiments where BODIPY-vancomycin was used as a fluorophore, the cytoplasm of the bacterial cells showed intense staining compared with Nile Red minimal intensity. Besides, BODIPY-vancomycin seems to label the cell wall and/or cell membrane. From these observations, we conclude that BODIPY-vancomycin binds not only to the outer part of the cell but also to higher extent to the MreB protein inside the cell.

**FIGURE 2:**
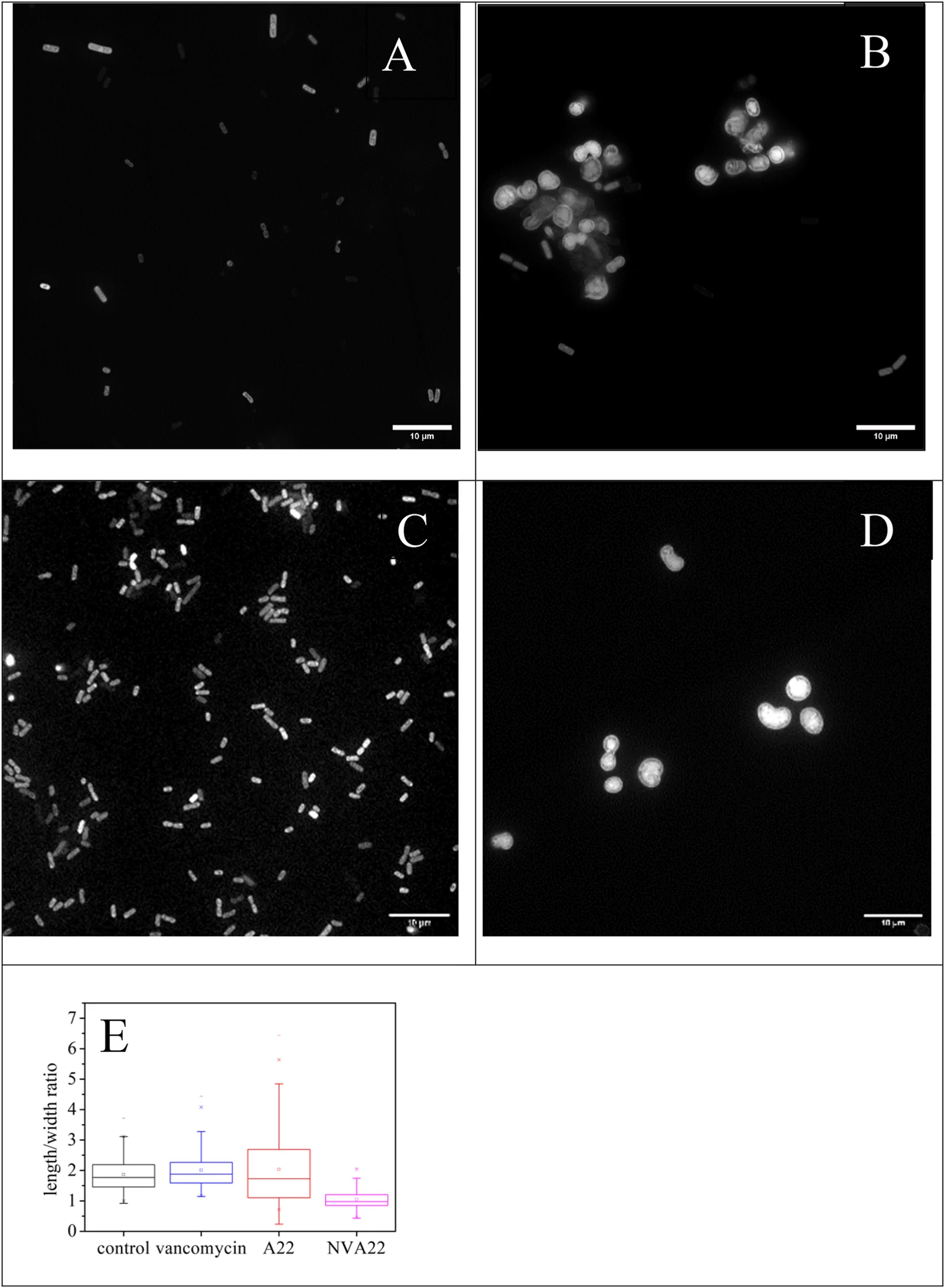
BODIPY-vancomycin-labeled *E.coli* cells. In all cases, the cells were labeled with BODIPY-vancomycin. *E. coli* K12 cells were labeled with BODIPY-vancomycin **(A)** in the absence of other antibiotics (A22 and vancomycin), and the rod shape of *E. coli* cells was observed. **(B)** In the presence of A22, most cells became round, although some rod-shaped cells were also observed. **(C)** When non-labeled vancomycin was added, the cells remained rod-shaped. **(D)** When non-labeled vancomycin and A22 were applied simultaneously, every cell lost its rod form. **(E)** Vancomycin enhanced the effect of A22. The round shape-forming effect of A22 is notably increased in the presence of vancomycin (A22 compared with vancomycin + A22) (n = 100–130).

#### 3.1.5 Determination of the shape of cells

A22 is a specific inhibitor of MreB. In its presence, MreB loses its function, resulting in round cells instead of rod-shaped ones. This effect depends on the concentration of A22 and the cell division phase. Applying the lowest concentrations of A22 (6.25 μg/mL) coming from our microbiological assay, some of the cells remained rod-shaped, while others were round (Figure 2B). Vancomycin treatment at the lowest concentration (1.56 μg/mL) did not influence the shape of the rod-shaped cells, which maintained their natural form (Figure 2C). However, when vancomycin and A22 are used simultaneously at the lowest concentrations (1.56 and 6.25 μg/mL, respectively), all cells take on a round shape (Figure 2D). This observation also supports the earlier experiences that vancomycin enhances the destabilizing effect of A22 on MreB, which can be considered a form of synergism. In addition, in the A22-containing samples (without and with vancomycin, Figures 2B,D), when a round shape of the cells develops, MreB seems to be delocalized from the membrane and appears with higher intensity in the cytoplasm. This is presumably why a gap appears between the cell wall lipid II signal and the cytoplasmic MreB signal. Furthermore, it is impressive that not only the shape of the cells has changed from rod-shaped to round, but also their volume has undergone significant growth. To analyze the length-to-width ratio (Figure 2E), the median and ratio distributions of the control cells refer to the typical rod-shaped cell population. In the presence of A22, the distribution of the length-to-width ratio is much wider, indicating that not only a rod-shaped but also a round-shaped population of the cells is also present; the heterogeneity of the cell population is clearly seen. In case of the presence of both A22 and vancomycin, the median is close to 1 (typical for spherical cells), and the distribution of the length-to-width ratio is narrow, indicating a more homogenic size and shape cell population; all the cells are close to round shape.

### 3.2 *In vitro* measurements on *Tm*-MreB and vancomycin complex formation

#### 3.2.1 Fluorescence anisotropy measurements of BODIPY-vancomycin on *Tm-*MreB

To investigate direct vancomycin-MreB binding, we measured the steady-state fluorescence anisotropy and fluorescence intensity of BODIPY-vancomycin in the presence of the expressed *Thermotoga maritima*-MreB (*Tm*-MreB). The results of the steady-state anisotropy measurements show that BODIPY-vancomycin binds to *Tm-*MreB in a concentration-dependent manner (Figure 3A), and the calculated binding constant in buffer A (4 mM Tris–HCl, pH = 8.0, 0.1 mM CaCl_2_) was found K_d_ = 8.33 ± 0.57 μM. Further, we calculated the binding affinity of BODIPY-vancomycin to *Tm-*MreB under different buffer conditions (Supplementary Table S1). The binding constant in the presence of ATP (0.2 mM), high KCl (100 mM), and both of them slightly changed, K_d_ = 6.89 ± 0.51 μM, K_d_ = 5.59 ± 1.21 μM, and K_d_ = 4.05 ± 0.72 μM, respectively, meaning that binding is strongest when nucleotide is present and salt concentrations are closer to physiological conditions (Supplementary Table S1).

**FIGURE 3:**
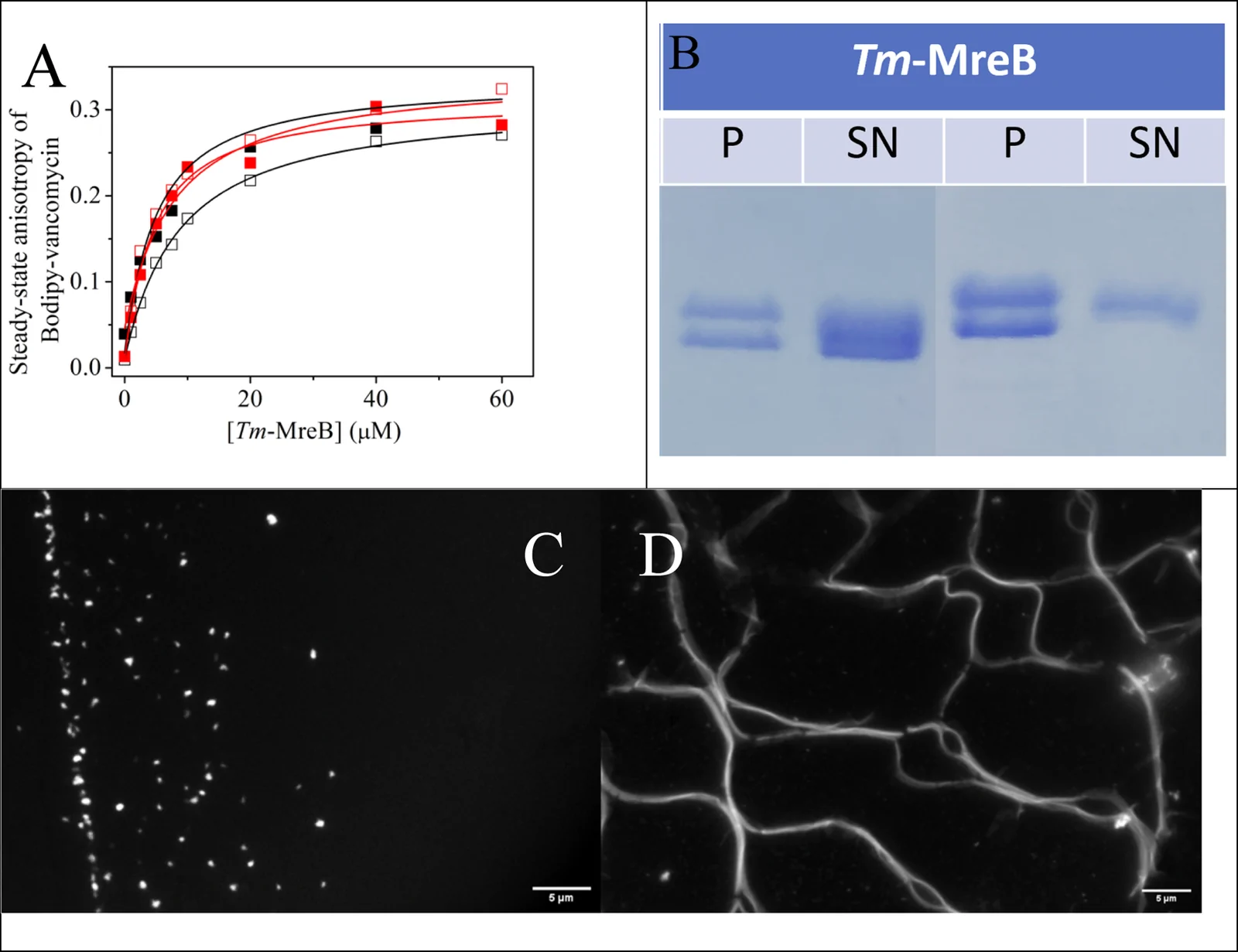
Vancomycin binds directly to *Tm*-MreB and forms bundles. **(A)** *Tm*-MreB binding increases the steady-state anisotropy of BODIPY-vancomycin; 1 μM BODIPY-vancomycin was incubated with *Tm*-MreB in various concentrations, and the steady-state anisotropy of each sample was measured. Black squares (buffer A), red squares (buffer A in the presence of polymerization salt), filled squares (in the presence of 0.2 mM ATP), and unfilled squares (in the absence of ATP). BODIPY-vancomycin binds to *Tm*-MreB in any buffer conditions with similar affinities. **(B)** Vancomycin stabilizes interfilamental connections between adjacent MreB molecules. Vancomycin (NV = non-labeled vancomycin) tends to form MreB bundles that can be sedimented by ultracentrifugation. Therefore, the amount of *Tm*-MreB increased in the pellet (P) in the presence of NV. **(C,D)** BODIPY-vancomycin labels *Tm*-MreB and native vancomycin forms MreB bundles in fluorescence microscope BODIPY-vancomycin-labeled *Tm*-MreB filaments in the absence **(C)** or presence **(D)** of vancomycin.

The direct binding between the *Tm*-MreB protein and vancomycin was also confirmed by our additional spectroscopic measurements, such as (i) the shift in the fluorescence emission maximum of BODIPY-vancomycin upon *Tm*-MreB binding (Supplementary Figure S2), (ii) the increment of the steady-state anisotropy value of BODIPY-vancomycin upon the addition of non-labeled vancomycin (Supplementary Figure S3), and (iii) the change in the fluorescence of *Tm*-MreB tryptophan upon the addition of non-labeled vancomycin (Supplementary Figure S4).

#### 3.2.2 SDS-PAGE measurements of the binding of BODIPY-vancomycin on *Tm-*MreB

To investigate whether vancomycin can induce a conformational change in *Tm*-MreB that alters the filament structure, sedimentation experiments were performed. *Tm*-MreB (20 μM) was incubated overnight with vancomycin at a concentration of 150 μg/mL. *Tm*-MreB in the presence and absence of vancomycin (Figure 3B) was centrifuged and analyzed by SDS-PAGE. A significant increase in the amount of pellet (P) was observed in the presence of vancomycin (Figure 3B), indicating that vancomycin binding strengthens the intermolecular connection between *Tm-*MreB monomers, without knowing the exact intermolecular and/or intramolecular modifications.

#### 3.2.3 Fluorescence microscopy measurements of the binding of BODIPY-vancomycin on *Tm-*MreB

Under SIM, the structural differences between the *Tm-*MreB (20 μM) saturated with vancomycin (100 μg/mL) and *Tm*-MerB alone were compared. To visualize the structure of the filaments, 1 μg/mL BODIPY-vancomycin was applied (Figures 3C,D). The images clearly show a remarkable structural difference ascribed to the effect caused by vancomycin binding. This finding confirms the results of the sedimentation experiment.

### 3.3 Docking calculations support binding between vancomycin and *Tm*-MreB

To support our experimental results, we performed *in silico* docking calculations. Both the monomeric and dimeric forms of vancomycin were docked to a representative unit of the *Tm-*MreB filament (target). The target was either a monomeric or a dimeric form of *Tm-*MreB. The search area included both the dimerization interface (Ent et al., 2001) and the ATP-binding pocket (Ent et al., 2001). One hundred docking runs were performed in all cases, and they resulted in two main sites located in the dimerization interface (Figure 4). The corresponding target–ligand interaction energy (E_inter_) values were calculated and are listed in Supplementary Table S3.

**FIGURE 4:**
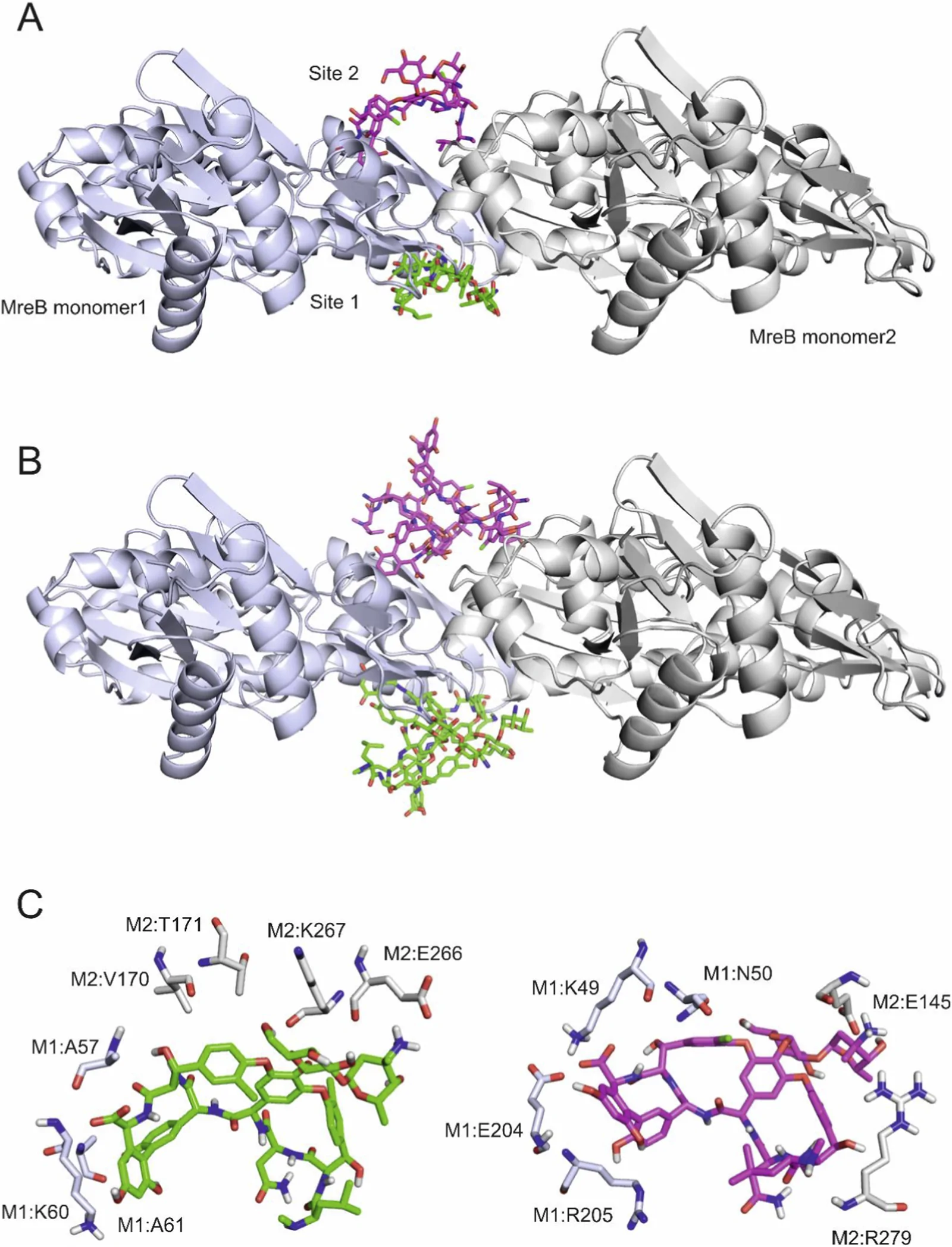
The vancomycin molecule has two binding sites on MreB. **(A)** The structure of monomeric vancomycin molecules bound (sticks, rank1: green, rank 22: magenta) to Site 1 (down) and Site 2 (up) of the dimeric MreB filament (cartoon, monomer1: blue–white, monomer2: gray). **(B)** The structure of dimeric vancomycin molecules bound (sticks, rank1: magenta. rank2: green) to Site 1 (down) and Site 2 (up) of the dimeric MreB filament (cartoon, monomer1: blue–white, monomer2: gray). **(C)** Close-up view of monomeric vancomycin bound to Sites 1 and 2 of the dimeric MreB filaments, showing the target residues interacting with vancomycin (sticks, on Site 1: green, on Site 2: magenta) within 3.5 Å distance.

The monomeric form of vancomycin tends to dock to Site 1 with the best E_inter_ for both the monomeric and dimeric forms of *Tm-*MreB. Docking calculations with monomeric and dimeric *Tm-*MreB also revealed the same binding conformations of monomeric vancomycin at Sites 1 and 2; however, they were not the best energy poses on the dimeric *Tm-*MreB (Supplementary Tables S2 and S5). As Figure 4 illustrates, vancomycin may play a stabilizing and bridging role between *Tm-*MreB monomer units. A close-up view illustrates that at Sites 1 and 2, the main stabilizing interactions in vancomycin positions of the best E_inter_ include H-bonds with target residues M1:A61 and M2:E266 at Site 1 and M1:K49 and M2:E145 at Site 2 (Figure 4C) that may be good candidates for future site-directed mutagenesis experiments.

At the same time, the dimeric vancomycin prefers Site 2 and shows a comparably good E_inter_ as observed at Site 1 (Supplementary Table S2). However, vancomycin still forms a bridge between the monomer *Tm-*MreB units, and therefore, it could maintain its stabilizing role (Figures 4 and 5). To quantify the stabilizing role of vancomycin, a stabilizing energy contribution of vancomycin was calculated (E_st_, Equation 3). E_st_ accounts for the energy gain corresponding to the presence of vancomycin during *Tm-*MreB dimerization (Figure 5; Supplementary Table S3). Binding of both vancomycin monomeric and dimeric forms increased E_st_ between the monomeric *Tm-*MreB units by at least 27% (Figure 5A). Therefore, it can be concluded that vancomycin may enhance *Tm-*MreB dimerization (Figure 5B).

**FIGURE 5:**
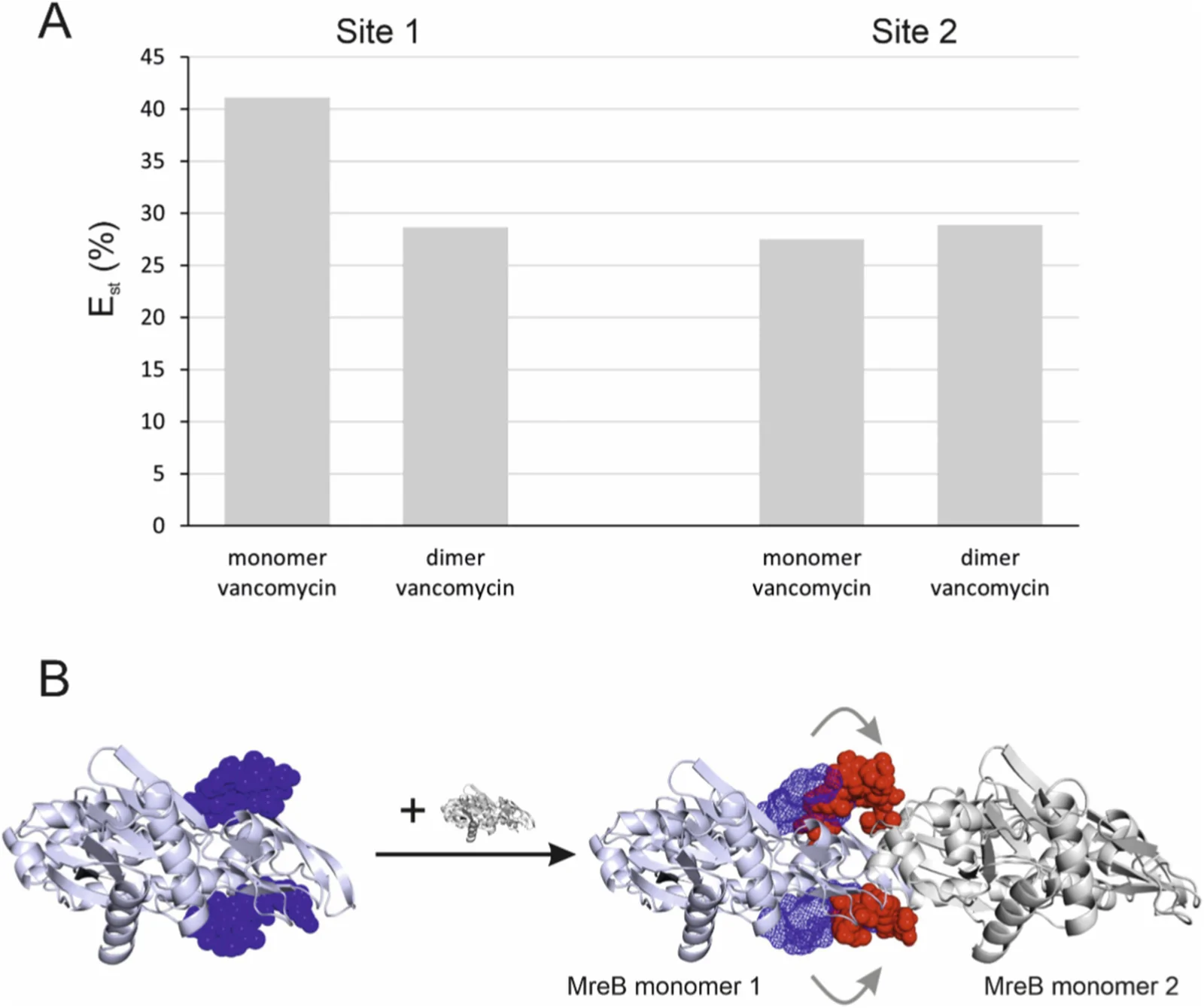
**(A)** The stabilization effect of vancomycin on the MreB–MreB interaction expressed as Est (Equation 3, Methods) at Sites 1 and 2. **(B)** The role of vancomycin in assisting MreB dimerization. Monomeric vancomycin (blue, spheres) bound to Sites 1 and 2 of MreB monomer1 (blue–white, cartoon) may assist the linking of MreB monomer2 (gray, cartoon) and move to a bridging position (red, spheres) to stabilize the dimerized MreB units. Gray arrows represent the relocation and repositioning of monomeric vancomycin.

A22 has an experimental complex structure with ADP and MreB (*Caulobacter vibrioides*). MreB sequences of *Thermotoga maritima* and *Caulobacter vibrioides* are 52% identical the RMSD of the ADP, and A22-binding sites show minimal difference, 0.668 and 1.652 Å, respectively. Vancomycin-binding sites (neither Site 1 nor Site 2) did not overlap with either A22 or ADP (nucleotide) binding pocket after the superposition of the MreB–ADP–A22 complex onto the MreB-vancomycin structure. This finding supports our experimental results that vancomycin can bind to MreB even in the presence of both ADP and A22, so there is no structural barrier or obstacle for the synergistic effect of A22 and vancomycin on MreB function.

The synergistic effect of the two substances was thus confirmed in three ways: inhibition of cell proliferation, drastic reduction in the minimal inhibitory concentration, and changes in cell morphology.

## 4 Discussion

Over the last couple of decades, the danger of bacterial infections has intensified because of the rapid increase in antibiotic resistance. The widespread use of antibiotics and the constant modification of antibacterial agents have failed to yield a breakthrough; therefore, discovering new antimicrobial pathways is crucial. The bacterial cell wall has always been one of the primary targets in the combat against pathogens, as bacteria quickly become non-viable in its absence. The MreB filamentous system, located beneath the cell membrane, plays a primary role in cell wall assembly and an essential role in directing peptidoglycan synthase localization and cell wall synthesis. Due to its function of maintaining cell shape, extensive research is being conducted on the MreB protein and its binding partners, especially on those that can inhibit the primary function of the protein.

A promising way is to combine inhibitory agents whose synergistic interaction can result in a more powerful antibacterial effect against pathogenic bacteria. In this paper, we investigated the combined effect of the MreB-specific antimicrobial A22 and vancomycin, a widely used antibiotic.

Vancomycin is an effective antibiotic against Gram-positive bacteria, such as *Enterococcus* and *Streptococcus* species, which are members of the ESKAPEE group of pathogens. The members of this group can “escape” commonly used antibiotics thanks to their increasing multidrug resistance. The MIC values for these pathogens range from a few μg/mL to a few tens of μg/mL. Vancomycin is not effective against Gram-negative bacteria, and its MIC value (200 μg/mL) is significantly elevated. However, in the case of combined use of vancomycin and A22—a known small molecule inhibitor of the MreB protein—the MIC value decreases dramatically to 1.56 μg/mL, a value characteristic of Gram-positive bacteria. This result confirms that the antimicrobial efficacy of vancomycin can be extended to Gram-negative bacteria when administered together with A22.

To investigate the effect of vancomycin on the cell shape and intracellular distribution of MreB filaments, A22 combined with fluorescently labeled BODIPY-vancomycin was used. In our observation, BODIPY-vancomycin appears not only beneath the cell wall according to the fluorescent microscopy measurements, but the shape of the cells changes from rod-like to spherical. This means that in the presence of A22, the entry of vancomycin into the *E. coli* cell is strongly promoted. The exact mechanism by which vancomycin can enter *E. coli* is not known yet; the change in the structure of the cell wall caused by A22 can facilitate the entrance. This finding leads us to the conclusion that A22 and vancomycin have a synergistic interaction with each other, meaning that their combined antibacterial effect increases considerably by low applied concentrations. It can be concluded that vancomycin treatment can also be extended to infections caused by not only Gram-positive but also Gram-negative bacteria without increasing the concentration of the antibiotic, when used in combination with low-concentration A22. MreB-vancomycin binding can occur at serum vancomycin concentrations developed during antibiotic treatment.

As previously described, vancomycin is unable to penetrate the membranes of *E. coli* cells. However, in the presence of A22, vancomycin finds its way into the intracellular space and, according to our observations, binds directly to the MreB filamentous network beneath the cell wall. A22 diffuses into the cell and disrupts cell wall synthesis and other cellular functions, resulting in cell death. The combined application of A22 and vancomycin enhances this progression, suggesting a strong synergistic interaction between them. Direct binding of vancomycin to MreB filaments can cause intrafilamental and/or interfilamental structural changes, as confirmed by fluorescence anisotropy and intensity measurements. In addition to its clinical relevance, fluorescently labeled vancomycin-MreB (in our study BODIPY-vancomycin-MreB) offers technological advantages, as it significantly facilitates the visualization of the spatial distribution of the MreB protein within the cell. Although our data clearly show the synergistic interaction of A22 and vancomycin on the MreB monomer/filament, this question needs further investigation.

Using *in silico* docking experiments, we aimed to identify the binding sites of vancomycin on MreB filaments to elucidate whether the binding sites of A22, the nucleotide, and vancomycin interfere with one another. A22 is known to inhibit the function of MreB by stopping the nucleotide hydrolysis by blocking the inorganic phosphate (P_i_) release. In solution, there is an equilibrium between the monomeric and dimeric forms of vancomycin. Docking calculations clarified that both forms of vancomycin can interact with the modeled MreB dimer at distinct binding sites (neither Site 1 nor Site 2). In both cases, vancomycin was shown to adopt a bridging position, thereby stabilizing the filament. The binding sites of A22 and ADP have been experimentally determined for MreB from *Caulobacter vibrioides*. Superposition of the structures of MreB *Thermotoga maritima* and *Caulobacter vibrioides* vancomycin-binding sites (neither Site 1 nor Site 2) do not overlap with the A22 or nucleotide-binding pockets. These well-separated binding sites allow A22 and vancomycin to act simultaneously on the MreB filament. These spatially distanced binding sites provide a structural basis for their synergistic effect.

Our results are consistent with the currently emerging trend that the synergism between two or more drug molecules may be a potential solution to the urgent problem of antibiotic resistance.

## Data Availability

The raw data supporting the conclusions of this article will be made available by the authors, without undue reservation.
