# Supplementary material for: Bacterial actin MreB as a new antibiotic target
Source: Front Mol Biosci. 2026 Oct 1;13:1912759. doi: 10.3389/fmolb.2026.1912759 (PMC13669091; doi:10.3389/fmolb.2026.1912759)
Supplement: Supplementary file 1 [file DataSheet1.pdf]

# *Supplementary Material*

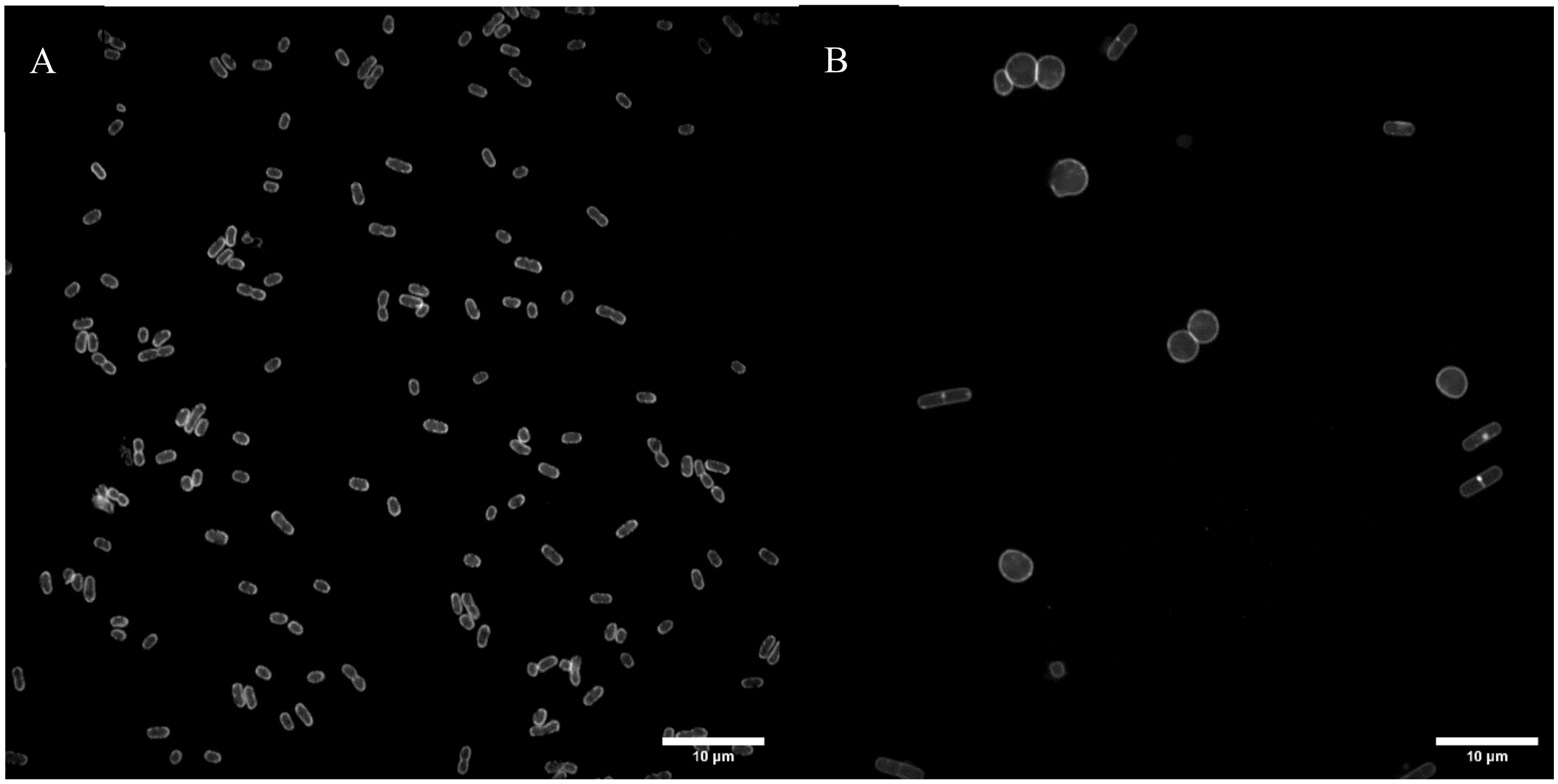


1. **Membrane labelling of *E.coli* K12 cells with Nile Red**

Figure S1.:*E. coli* K12 cells were labelled with Nile Red (10 µg/ml, membrane labelling) in the absence (A) or presence (b) of A22.

2. **Determination of the binding constants of Bodipy-vancomycin and Tm-MreB under different buffer conditions by fluorescence anisotropy measurements**

| Condition | Kd |
|---|---|
| Buffer A | 8.33 ± 0.57 µM |
| Buffer A + 0.2 mM ATP | 6.89 ± 0.51 µM |
| Buffer A + 100 mM KCl, 2 mM $MgCl_2$ | 5.59 ± 1.21 µM |
| Buffer A + 100 mM KCl, 2 mM $MgCl_2$ + 0.2 mM ATP | 4.05 ± 0.72 µM |

*Table S1. The affinity of Bodipy-vancomycin to Tm-MreB depends on buffer conditions. The strongest binding can be detected in conditions that are closest to physiological environment in bacterial cells. (Buffer A: 4 mM Tris-HCl, pH = 8.0, 0.1 $CaCl_2$)*

3. **Fluorescence intensity measurements of Bodipy-vancomycin upon *Tm*-MreB binding**

Another proof of binding of vancomycin and *Tm*-MreB is a fluorescence intensity measurement. The fluorescence intensity of constant concentration (1 µM) of Bodipy-vancomycin was measured in the presence of 0-60 µM concentration of *Tm*-MreB. The illumination wavelength was set to 503 nm, the

emission peak shifted upon complex formation from 511 nm to 516 nm (red shift) in a concentration-dependent manner (Figure S2). This measurement also supports our assumption, that there is a direct binding between vancomycin and *Tm*-MreB. The calculated $K_d = 2.12 \pm 0.65$ µM which is relatively close to the results coming from the fluorescence anisotropy measurements.

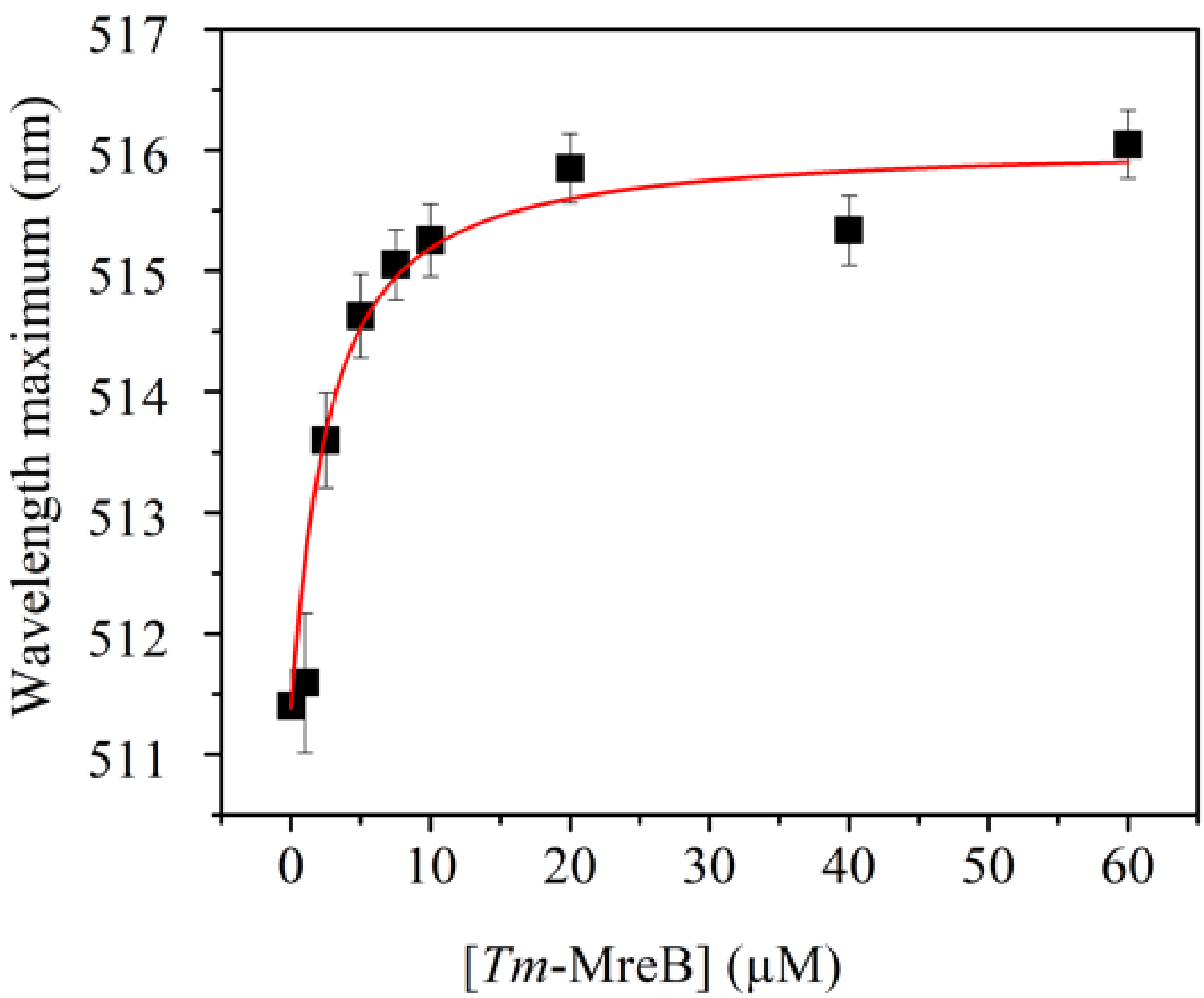


***Figure S2. The fluorescence emission of Bodipy-vancomycin shows a red shift upon Tm-MreB binding.*** *1 µM Bodipy-vancomycin was incubated with Tm-MreB in different concentrations in buffer A and the fluorescence emission maxima were plotted as a function of Tm-MreB concentration. The Bodipy-vancomycin is sensitive for the change of environment, showing Tm-MreB binding.*

4. **Complex formation between Bodipy-vancomycin and native vancomycin upon *Tm*-MreB binding**

We also investigated whether Bodipy-vancomycin and native vancomycin share the same binding site on the *Tm*-MreB molecule. To achieve this, we designed an inhibitory assay. However, our data led to a very interesting observation, which is already somewhat known for vancomycin. We observed that the anisotropy of the Bodipy-vancomycin-*Tm*-MreB complex was slightly increased by adding of native vancomycin (Fig. S3). This is explained by the fact that vancomycin tends to dimerize and contribute to steady-state anisotropy. The fact that it does happen in the presence of *Tm*-MreB is explained by the fact that native vancomycin also binds to *Tm*-MreB, promoting dimerization and the formation of higher-order oligomers, or by forming heterodimers between Bodipy-vancomycin and native vancomycin. This supports the previously predicted *in silico* results (38).

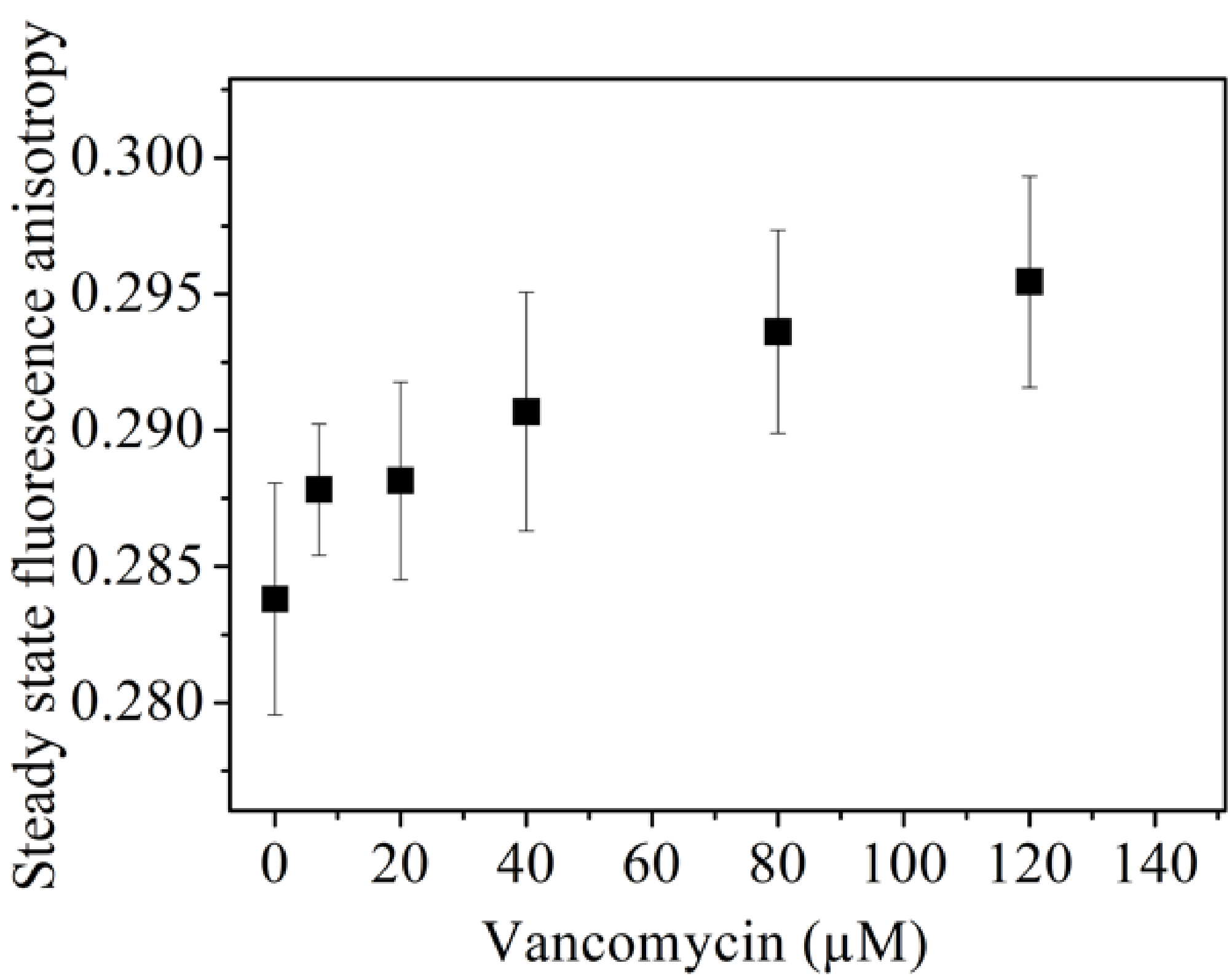


***Figure S3. Native vancomycin binds to Bodipy-vancomycin-Tm-MreB complex.*** *1 μM Bodipy-vancomycin was incubated with 60 μM of Tm-MreB in buffer A and the fluorescence anisotropy of Bodipy-vancomycin was measured in the presence of increasing vancomycin concentrations. The steady-state anisotropy of Bodipy-vancomycin increases when unlabelled vancomycin binds to the Tm-MreB-Bodipy-vancomycin complex.*

## 5. **Tryptophan fluorescence emission changes upon vancomycin binding**

In order to experimentally prove the *in silico* data, additional experiments were completed. The E145 and E266 amino acids are located near to the only tryptophan of *Tm*-MreB (W172, Figure 4, yellow spheres) and based on calculations the neighbouring V170 and T171 can also be binding amino acids (Table S4). In our experiments the fluorescence emission of the tryptophan of *Tm*-MreB was measured in the presence of vancomycin. We observed that the fluorescence emission of the tryptophan dropped by low vancomycin concentration (Figure S4). This is confirmed by our steady state anisotropy data, showing binding of vancomycin to *Tm*-MreB with few micromolar affinity (Table S1). With a further increase of vancomycin concentration, the fluorescence emission increases consistently, which may represent a significant conformational change in the environment of tryptophan. A blue shift of a few nanometres can be also measured in tryptophan emission (data not shown). Therefore, we can conclude that the conformation of *Tm*-MreB is altered upon binding of vancomycin, and the region around the tryptophan is involved in bond formation.

The other position that is calculated as a binding site is the area around A61 and K49. In the structurally homologue actin, this region is called the D-loop. Interestingly, this region is also referred to as the molecular linker, as its conformation essentially determines intermolecular binding (39). Since we have found that MreB monomers or oligomers are organized into larger structures for vancomycin binding (Figure 3D), we can speculate that this part may play a similar role for *Tm*-MreB. Thus, by binding to the D-loop of *Tm*-MreB, vancomycin may be able to enhance the binding of MreB molecules to each other. Our centrifugation-based assay also supported that vancomycin increases the amount of MreB in the sediment, thus presumably increasing the number of filaments and MreB bundles formed (Figure 3B and D).

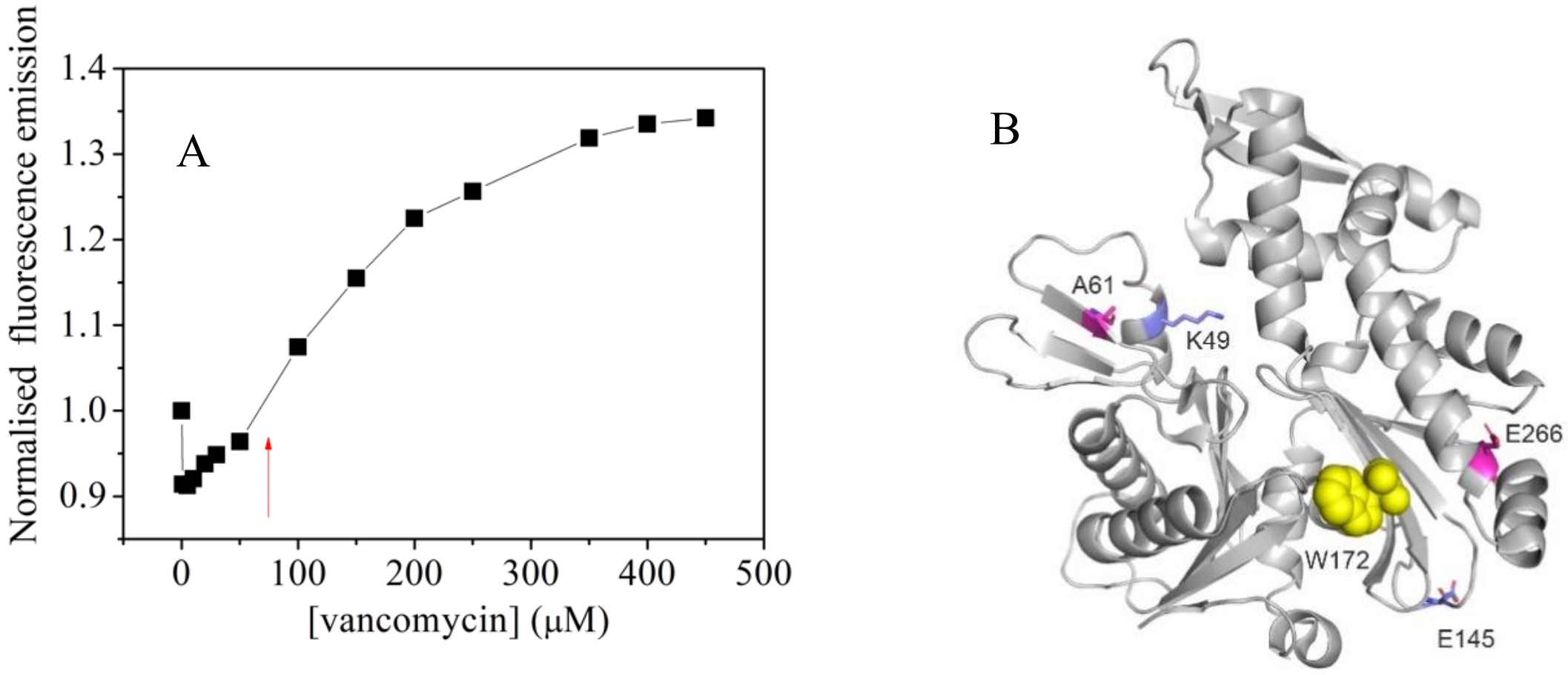


***Figure S4: Localisation of tryptophan on Tm-MreB and the effect of vancomycin binding. A: Vancomycin affects intrinsic fluorescence of Tm-MreB****. The fluorescence emission of tryptophan with 20 μM Tm-MreB was measured in buffer A and increasing vancomycin concentrations. Next, the fluorescence emission maxima were divided by the emission intensity of the vancomycin-free sample. The tryptophan emission shows high sensitivity for vancomycin binding because at the lowest applied concentration (by 1μM vancomycin) there was a drop observed in the fluorescence emission. By adding*

*more vancomycin, the fluorescence intensity of tryptophan residue tends to increase. The red arrow indicates the concentration of vancomycin that was applied in in vivo measurement. Large excesses of vancomycin can bind to Tm-MreB, which indicate complex formation.* ***B: The structure of Tm-MreB with the amino acids that are stabilizing the vancomycin binding (sticks in magenta: Site 1, sticks in blue: Site 2) and the tryptophan (yellow spheres)****. Based on molecular docking calculations, it can be hypothesized that there are two binding sites on a Tm-MreB monomer located on the opposite site of the molecule. These binding sites appear to be responsible for the bundling activity of vancomycin. Based on the in silico results, the A61 and E266 amino acids in Site 1, and K49 and E145 in Site 2 are involved in vancomycin binding to MreB.*

**SI. 2. MD data**

| | Monomeric vancomycin to monomeric MreB | Monomeric vancomycin to dimeric MreB | Dimeric vancomycin to dimeric MreB |
|---|---|---|---|
| Site 1 | Rank 1/58<br>-13.66 | Rank 1/55<br>-15.14 | Rank 2/2<br>-10.50 |
| Site 2 | Rank 28/58<br>-10.18 | Rank 22/55<br>-10.37 | Rank 1/2<br>-10.00 |

**Table S2.** Ranking of docking results based on the intermolecular interaction energies ($E_{inter}$, kcal/mol) of the ligands. The first number represents the best energy docked conformation on Site 1 and Site 2, and the second number shows the sum of all ranks docked after 100 docking runs.

| Two monomeric apo MreBs ($E_{dimer}$(apo)) | Site 1 ($E_{dimer}$(holo)) | | Site 2 ($E_{dimer}$(holo)) | |
|---|---|---|---|---|
| | Monomeric vancomycin | Dimeric vancomycin | Monomeric vancomycin | Dimeric vancomycin |
| -16.14 | -22.78 | -20.76 | -20.58 | -20.80 |

**Table S3.** Raw data of column diagram (Fig. 5A). Dimerization intermolecular interaction energy values calculated for the ligand-free dimeric MreB units. $E_{dimer}$(apo) and $E_{dimer}$(holo) (vancomycin-complexed, dimerized MreBs) were constructed using the representative docked conformations at the binding sites obtained from each docking calculation. Dimerization intermolecular interaction energy includes van der Waals, H-bond, desolvation, and electrostatic interaction energies. The dimension of energy values is kcal/mol.

| | Target residues | Site1 | | Site2 | |
|---|---|---|---|---|---|
| | | Rank2 | Rank1 | Rank52 | Rank22 |
| Monomer 1 | ASN14 | | | x | |
| | ASN27 | | | x | |
| | GLU28 | | | x | |
| | PRO29 | | | x | |
| | LEU46 | | | x | |
| | LYS49 | | | x | x |
| | ASN50 | | | | x |
| | GLY53 | x | | | |
| | THR55 | | x | | |
| | PRO56 | | x | | |
| | ALA57 | | x | | |
| | LYS60 | x | x | | |
| | ALA61 | x | x | | |
| | ILE62 | x | | | |
| | ARG63 | x | | | |
| | ARG66 | x | | | |
| | ASP67 | x | | | |
| | GLU181 | x | | | |
| | GLU184 | x | | | |
| | VAL187 | x | | | |
| | ALA197 | x | x | | |
| | ILE198 | x | x | | |
| | GLY199 | x | | | |
| | GLU200 | x | | | |
| | ARG201 | | | | x |
| | GLU204 | | | | x |
| | ARG205 | | | x | x |
| | ILE208 | | | x | |
| | GLU209 | | | x | |
| | LEU312 | | | x | |
| Monomer 2 | GLU145 | | | | x |
| | SER147 | | | | x |
| | VAL170 | | x | | |
| | THR171 | | x | | |
| | GLU266 | | x | | |
| | LYS267 | | x | | |
| | PRO269 | x | x | | |
| | PRO270 | x | x | | |
| | ASP275 | | | | x |
| | GLU278 | | | | x |
| | ARG279 | | | | x |

**Table S4.** Dimeric MreB residues interacting with the docked representative monomeric vancomycin within 3.5 Å distance on Site 1 and Site 2.

| | Monomeric vancomycin to monomeric MreB | Monomeric vancomycin to dimeric MreB |
|---|---|---|
| Site 1 | Rank 1<br>-13.66 | Rank 2<br>-14.77 |
| Site 2 | Rank 40<br>-9.22 | Rank 52<br>-8.08 |

**Table S5.** Energy and rank number of monomeric vancomycin on Site 1 and Site 2 of monomeric and dimeric MreB docked with the same conformation.
